# N-acetylcysteine reduces severity and mortality in COVID-19 patients: A systematic review and meta-analysis

**DOI:** 10.5455/javar.2023.j665

**Published:** 2023-06-30

**Authors:** Mohammad Shah Alam, Mohammad Nazmol Hasan, Zannatul Maowa, Fahima Khatun, K. H. M. Nazmul Hussain Nazir, Mohammad Zahangeer Alam

**Affiliations:** 1 Department of Anatomy and Histology, Bangabandhu Sheikh Mujibur Rahman Agricultural University, Gazipur, Bangladesh; 2 Department of Statistics, Bangabandhu Sheikh Mujibur Rahman Agricultural University, Gazipur, Bangladesh; 3 Department of Pathobiology, Bangabandhu Sheikh Mujibur Rahman Agricultural University, Gazipur, Bangladesh; 4 Department of Microbiology and Hygiene, Bangladesh Agricultural University, Mymensingh, Bangladesh; 5 Department of Environmental Science, Bangabandhu Sheikh Mujibur Rahman Agricultural University, Gazipur, Bangladesh

**Keywords:** N-Acetylcysteine, COVID-19, SARS-CoV-2, severity, mortality, treatment

## Abstract

**Objectives:**

Recent clinical studies suggest that oxidative stress is one of the key players in the pathogenesis of coronavirus disease 2019 (COVID-19), and N-acetylcysteine (NAC), a potent antioxidant, has been shown to improve clinical outcomes in COVID-19 patients. We conducted a systematic review and meta-analysis of the literature published on the therapeutic intervention of NAC on COVID-19 infection.

**Methods:**

We searched PubMed, Google Scholar, and Science Direct. We identified and screened eight studies with 20,503 participants, including 2,852 in the NAC-treated group and 17,651 in the placebo group, which reported the effect of NAC on COVID-19 infection. A meta-analysis was performed using forest plots under fixed effect estimates based on the standardized mean difference (SMD) and risk ratio (RR).

**Results:**

Pooled analysis showed that NAC was associated with lower mortality in patients with COVID-19 compared with the placebo group [RR, 0.65; (95% CI: 0.56 to 0.75); *p* < 0.0001]. Similarly, C-reactive protein (CRP) [SMD, −0.32; (95% CI: −56 to −0.09); *p* = 0.0070] and D-dimer [SMD, −0.35, (95% CI: −0.59 to −0.10; * p =* 0.0062] levels were significantly decreased, and the oxygenation marker, PaO_2_/FiO_2_ ratio, was increased in the NAC-treated group compared with the placebo group [SMD, 0.76; (95% CI: 0.48 to 1.03); *p* < 0.0001].

**Conclusion:**

Although the number of included studies was minimal, this meta-analysis suggests that NAC may have a positive effect on COVID-19 outcomes, specifically, a significant decrease in CRP and D-dimer levels and a significant increase in oxygen saturation, which decreased mortality. We have also presented a comprehensive review of the role and mechanisms of NAC in patients with COVID-19.

## Introduction

COVID-19, caused by severe acute respiratory syndrome coronavirus-2 (SARS-CoV-2), causes complications in humans ranging from asymptomatic to severe pneumonia, heart attack, and multiorgan failure [1,2]. Although most COVID-19 patients (over 80%) have mild to no symptoms, approximately 14%–15% exhibit moderate symptoms of pneumonia characterized by acute lung injury, cough, and fever [3,4]. Furthermore, about 5% eventually develop severe illnesses characterized by acute respiratory distress syndrome (ARDS) and septic shock. The virus initially infects the epithelium of the upper respiratory tract and gradually spreads to the lower respiratory tract, resulting in a severe lung infection. The spike (S) of the virus binds to the cell membrane via angiotensin-converting enzyme 2 (ACE2) to initiate the viral entry process. Subsequently, transmembrane serine protease type 2, manifested in human cells, assists the virus’s entry into cells by proteolytic cleaving of the S protein [5]. Apart from the respiratory epithelial cells, ACE2 is also significantly expressed in the endothelium of the heart, kidney, intestine, testis, and ovary, leading to systemic transmission to other organs [6]. The binding of ACE2 to SARS-CoV-2 has been shown to downregulate ACE2, which may disrupt the conversion of angiotensin (Ang) II to Ang 1–7 [7]. Ang II is a potent stimulator of nicotinamide dinucleotide phosphate (NADPH) oxidase and increases superoxide (O_2_^•−^) production [8]. In contrast, Ang 1–7 blocks O_2_^•−^ production, thus, maintaining the balance of reactive oxygen species (ROS) in our body. Inhibition of ACE2 decreases Ang 1−7 synthesis and increases Ang II concentration, which enhances oxidative stress. Furthermore, SARS-CoV-2 increases the neutrophil/lymphocyte ratio, increasing ROS production through the NADPH oxidase pathway [9]. Many critically ill COVID-19 patients have low glutathione (GSH) and thiol levels [10,11]. In contrast, higher levels of ROS resulted in greater redox status (ROS/GSH ratio) and oxidative stress [10–12].

A dire medical condition in severe COVID-19 patients, called “cytokine storm syndrome,” is characterized by an abnormal inflammatory response associated with high levels of pro-inflammatory cytokines and neutrophils [13,14]. Pro-inflammatory cytokines, in particular elevated levels of C-reactive protein (CRP), D-dimer, and interleukin (IL) 1β, IL6, IL8, IL17, and tumor necrosis factor α (TNF-α), are associated with the worst outcome and high mortality in COVID-19 patients [13–15]. Such medical conditions are often found in patients with underlying comorbidities such as diabetes, cardiovascular disease, obesity, and immunosuppressive states. [13,16–18].

Oxidative stress and cytokine storms induce endothelitis and endothelial cell swelling in severe COVID-19 patients [13]. Endotheliitis activates the blood clotting process, factors II, V, VIII, IX, and X, and elicits von Willebrand factor (vWF) in endothelial cells. They also activate the platelets interacting with neutrophils and induce neutrophil extracellular trap (NET) formation [13]. NET generates thrombin and fibrin, resulting in vascular thrombosis that increases severity and mortality [19,20].

Therefore, oxidative stress combined with cytokine storms is now recognized as a key contributor to COVID-19 pathogenesis, severity, and mortality [9,21]. Since oxidative stress is associated with the severity of COVID-19, such as acute pneumonia, ARDS, septic shock, and thrombosis, effective neutralization of ROS by antioxidants may be a fascinating strategy for treating COVID-19 [22].

Many approaches have been proposed to treat COVID-19, such as optimal supportive care, including oxygen with fluid administration, non-invasive mechanical ventilation for critically ill patients, and recently FDA-approved COVID-19 vaccines as preventive measures. Although severity and mortality rates have recently decreased to a certain extent due to COVID-19 vaccine administration, the disease still spreads rapidly worldwide. The virus has mutated, evolving into different stains/variants and escaping antibodies. Indeed, vaccines have variant-specific activity and pose challenges for preventing and controlling new variant-associated COVID-19 [23]. In addition to vaccines, it is indispensable to identify effective therapeutics targeting oxidative stress and cytokine storms that are expected to treat severe COVID-19 patients effectively. N-acetylcysteine (NAC), an FDA-approved classic drug, is known for its mucolytic effects. It stimulates GSH biosynthesis, a powerful antioxidant that mops up ROS such as H_2_O_2_, O_2_^•−^, and ^•^OH, and thus directly promotes scavenging and free radical detoxification [9]. NAC has anticoagulant and thrombolytic effects [24–26]. NAC modulates inflammatory activity, protects against thrombotic conditions, and exerts an antiviral effect [27]. Furthermore, NAC has been found to inhibit nuclear factor kappa β (NF-κβ) activation in influenza and respiratory syncytial virus [28]. Potential pharmacological effects make it a plausible treatment option for COVID-19.

NAC was associated with improved respiratory outcomes from pneumonia, ARDS, chronic obstructive pulmonary disease [29,30], and other organ complications [31]. Experimental data indicate that NAC enhances clinical outcomes in patients with COVID-19 [32,33]. Several randomized control (RC) and clinical trials of NAC have recently been conducted in patients with COVID-19 [34,35]. However, the benefits of this drug have not been consistent among studies. For example, an RC trial of NAC found significant increases in blood oxygen saturation and a decreased length of hospital stay [36]. Moreover, a large retrospective cohort study found positive results that significantly reduced mortality [34].

In contrast, the RCT treatment using NAC by de Alencar et al. [35] did not reduce mortality or intensive care unit (ICU) stay duration in patients with COVID-19. Therefore, we performed a meta-analysis to evaluate whether the treatment of NAC may benefit patients with COVID-19. In addition, a comprehensive review of the role and mechanisms of NAC in patients with COVID-19 is presented. This comprehensive review with meta-analysis will contribute to a better understanding of the effects of NAC and potential mechanisms that may pave the way for mitigating COVID-19-related complications and reducing mortality.

## Materials and Methods 

We performed a systematic review and meta-analysis of the included peer-reviewed research articles. A meta-analysis is an appropriate approach for this study because the clinical question regarding the pharmacological efficacy of NAC in the treatment of COVID-19 is novel and limited. We followed the preferred reporting items for systematic reviews and meta-analysis (PRISMA) 2020 statement and guidelines to report our review process and findings [37].

### Ethics committee approval

Data were collected from peer-reviewed published research papers involving human samples and were therefore exempt from ethics committee approval.

### Search strategies and data sources

All available published peer-reviewed research articles were retrieved from online electronic databases such as Google Scholar, PubMed, and Science Direct until January 2023 using keywords: (NAC OR NAC OR acetylcysteine) AND (severe acute respiratory syndrome coronavirus 2 OR SARS-CoV-2 OR COVID-19 OR severity OR mortality). We studied titles and abstracts to select relevant papers. All authors conducted an independent screening of titles and abstracts. Full-text articles are then evaluated for eligibility.

### Inclusion and exclusion criteria

We included studies that met all of the following criteria: 1) RC trials, observational studies, retrospective cohort studies, or cross-sectional studies measuring oxidative stress markers, CRP, D-dimer, etc., after NAC treatment in COVID-19 patients; 2) studies calculating the length of hospital stay, ICU stay, and mortality rate after NAC treatment in patients with COVID-19; 3) studies evaluating oxidative stress markers H_2_O_2_, O_2_^•−^, and ^•^OH; and antioxidant markers superoxide dismutase (SOD), glutathione peroxidase, and catalase in patients with COVID-19 after NAC therapy; 4) inclusion of COVID-19 as diagnosed by a WHO-recognized protocol or standard recognized criteria; or 5) studies that provided sample size (*n*) means and SDs; 6) there were no limitations on age, outpatient or inpatient setting, or severity of the disease.

We excluded studies that fulfill one of the following criteria: 1) abstracts; 2) conference papers; 3) not an original research article; 4) not peer-reviewed literature; 5) narrative and systemic review; 6) scoping review; 7) meta-analysis; and 8) dual publications.

### Data extraction

Data were extracted by three independent authors, with disagreements resolved by discussion. Information was extracted and recorded systematically according to a pre-designed form as follows: name of the first author, year of publication, study design, sample size, NAC dosing, CRP, D-dimer, PaO_2_/FiO_2 _ratio, duration of hospital and ICU stay, and mortality rate. Discrepancies (if any) were resolved through discussion by both authors.

### Quality and bias assessment of included studies 

According to the Newcastle-Ottawa Scale (NOS) standard specification, all authors independently evaluated the quality of the included studies using the following criteria: 1) definition of the case; 2) definition of controls; 3) selection of controls; 4) repetitiveness of cases; 5) comparability of cases and controls; 6) ascertainment of NAC exposure; 7) same method of ascertainment for cases and controls; and 8) non-response rate [38]. Each of the studies was assigned a score of 1 if the studies addressed quality features 1 to 4 and 6 to 7; otherwise, the score was attributed as zero (0). Nonetheless, studies were assigned a score of 2 for feature 5 if they adjusted for both age and confounders; if they adjusted for only age or confounders, the study was assigned a score of 1; otherwise, it would get a score of 0. Accordingly, a study can receive a maximum score of 9, if it meets all quality standards of the NOS (Table 1).

Bias assessment based on funnel plots required more than 10 studies, whereas we found 8 studies containing desirable data. Therefore, we used Egger’s test for the same purpose [39]. A *p*-value considered for publication bias is <0.05; otherwise, it is unbiased.

### Intervention and outcome

The intervention of NAC is defined as the NAC treatment of COVID-19. The controls were placebos. The outcomes were severity and mortality, where mortality was defined as clinically non-survivor/death and severity was defined as a clinically increased level of inflammatory markers, CRP, D-dimer, and ferritin, and decreased length of hospital and ICU stay and oxygenation (PaO_2_/FiO_2_ ratio). Pooled or summary effect estimates were reported as a risk ratio (RR) or standardized mean difference (SMD).

### Meta-Analysis

The therapeutic efficacy of NAC on COVID-19 severity and mortality has been evaluated in included studies using the data of CRP, D-dimer, ferritin, ICU stay, hospital stay, PaO_2_/FiO_2_, and mortality. In this aspect, the effect of NAC was meta-analyzed for each outcome using a forest plot under fixed effect estimates based on the SMD and RR for severity indices and mortality, respectively. Studies reporting severity indices and mortality data were meta-analyzed to observe the summary effects. A *p-*value of less than 0.05 (<0.05) was set to predict a significant summary effect of NAC between the treated and control/placebo groups.

The heterogeneity among studies was calculated by the statistic, tested by the statistic, and interpreted as less than 25%: no heterogeneity; 25% to 49%: low heterogeneity; 50% to 74%: moderate heterogeneity; and 75% or greater: high heterogeneity. A *p-*value of <0.05 was set to assess significant between-study heterogeneity. Meta-analysis was performed using the “meta” package in the R programming language software [40].

**Table 1.** A table summarizing NAC-treated studies determining study location, design, sample size, NOS score, and COVID-19 mortality and severity index.

Study no.	Authors	Study location	Sample size		COVID-19 mortality and severity index	Study design	NOS score	Citation
			NAC	Cont.				
1	de Alencar et al. [35]	Brazil	67	68	Mortality, CRP, D-dimer, Hospital stay, ICU stay, PaO_2_/FiO_2_ ratio	RC trial	9	[35]
2	Izquierdo et al. [34]	Spain	2,071	17,137	Mortality	Retrospective cohort study	8	[34]
3	Assimakopoulos et al. [41]	Greece	42	40	Mortality, CRP, D-dimer, Ferritin, PaO_2_/FiO_2_ ratio	Retrospective cohort study	9	[41]
4	Taher et al. [42]	Iran	47	45	Mortality, Hospital stay, ICU stay, PaO_2_/FiO_2 _ratio	Pilot study	8	[42]
5	Chen et al. [43]	USA	7	9	CRP, Ferritin, PaO_2_/FiO_2 _ratio	Retrospective cohort study	7	[43]
6	Ibrahim et al. [32]	USA	9	9	CRP, Ferritin	Observational study	7	[32]
7	Avdeev et al. [33]	Russia	24	22	Mortality, CRP, D-dimer, PaO_2_/FiO_2 _ratio	Case-control study	8	[33]
8	Faverio et al. [44]	Italy	585	321	Mortality	RC trial	8	[44]
Total			2,852	17,651				

### Literature sources for comprehensive reviews 

We searched the peer-reviewed research literature in online electronic databases such as PubMed, PMC, Google Scholar, Science Direct, and Scopus. The search terms used were “role of NAC in respiratory infections or effects of NAC in COVID-19 or NAC in SARS-CoV-2 infection or SARS-CoV-2 or NAC and immune response to COVID-19 or NAC and COVID-19 severity and NAC toxicity’.

## Results

**Figure 1.**
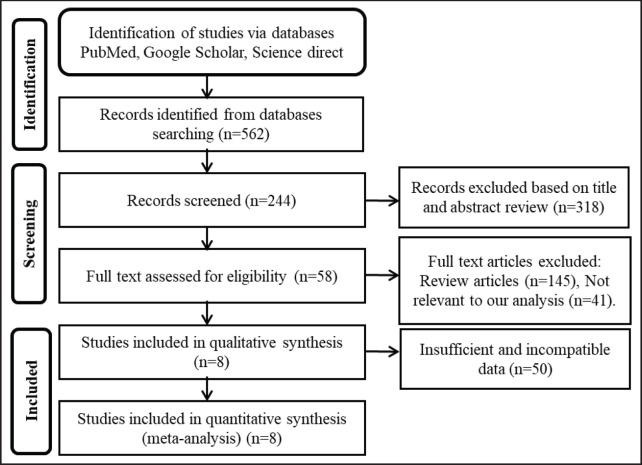
The PRISMA flow chart describes the number of studies identified, screened, and assessed for eligibility and included in the NAC data review process [adapted from [37]].

A search of online electronic databases specified in the search strategy yielded 562 unique peer-reviewed research articles (Fig. 1). After removing duplicates, 318 articles were excluded. After that, from the remaining 244 articles, 145 review articles, 41 non-relevant articles, and 50 articles with insufficient and incompatible data were excluded. Finally, eight studies—two RCTs, three retrospective cohort studies, one pilot study, one case-control study, and one observational study—met the inclusion criteria. They underwent a meta-analysis [32–35, 41–43].

### Features of the included studies

There were no remarkable differences in sex, age, comorbidities, or any other baseline features among all the included studies. There were 20,503 patients in the included studies, among whom 2,852 were in the NAC-treated group, and 17,651 were in the placebo group. The detailed features, including study location, study design, study size, COVID-19 severity index, and mortality of the included studies [32–35, 41–44], were illustrated in Table 1.

### Quality and bias assessment of included studies

According to the distributed score over the NOS quality standard features, two studies got a total score of 9, three studies got a total score of 8, and the rest got a total score of 7 (Table 1). We constructed forest plots to estimate the summary effect of NAC on COVID-19 severity indices and mortality using data on CRP, D-dimer, ferritin, duration of hospital and ICU stay, PaO2/FiO2, and the number of deaths. Concomitantly, we also examined the biases of the included studies according to the severity index and mortality of COVID-19 using Egger’s test. Egger’s test *p*-value was greater than 0.05 for all considered indexes, indicating that the studies were not biased for each index, although there was a high degree of heterogeneity (*I*^2^ = 59%–90%) (Table 2).

### Therapeutic efficacy of NAC in reducing the severity of COVID-19

**Figure 2.**
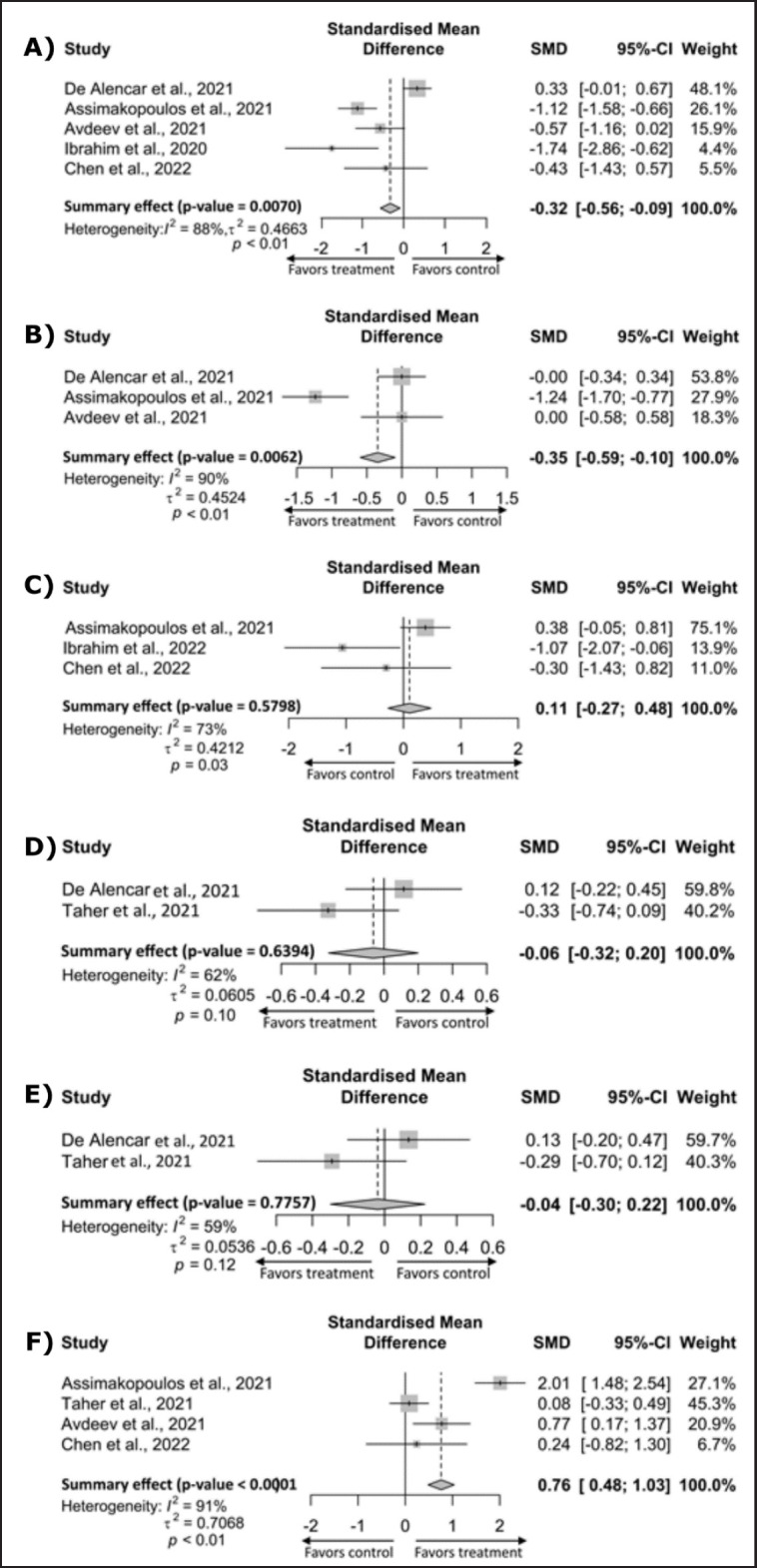
Forest plots for the severity index of COVID-19 in the NAC treatment group versus the placebo group. A) CRP, B) D-dimer, C) ferritin, D) hospital stay, E) ICU stay, F) PaO_2_/FiO_2_. CI, confidence interval; SMD, standardized mean difference.

The present meta-analysis evaluated the therapeutic efficacy of NAC on COVID-19 patients based on data on CRP, D-dimer, and ferritin, duration of hospital and ICU stays, and PaO_2_/FiO_2_ available in the included studies. Five of the studies contained data on CRP. The meta-analysis indicated that the NAC-treated group of COVID-19 patients had significantly lower CRP levels compared with the placebo group [SMD, −0.32; (95% CI: −56 to −0.09); *p-*value = 0.0070], although the studies have a high degree of heterogeneity (*I*^2^ = 88%) (Fig. 2A and Table 2). Four of the studies had data on D-dimer and oxygenation index (PaO_2_/FiO_2_ ratio), and pooled analysis showed that NAC treatment significantly reduced D-dimer levels in patients with COVID-19 compared to the placebo group [SMD, −0.35, (95% CI: −0.59 to −0.10); *p*-value = 0.0062], (*I*^2^ = 90%), (Fig. 2B). On the other hand, the PaO_2_/FiO_2_ ratio was significantly higher in the NAC-treated group of patients compared to the placebo group [SMD, 0.76; (95% CI: 0.48 to 1.03); *p-*value < 0.0001] (*I*^2^ = 59%) (Fig. 2F and Table 2). Two studies had data on hospital stays and ICU stays. However, the length of hospital stay [SMD, −0.06; (95% CI: −0.32 to 0.20); *p*-value = 0.6394] and ICU stay [SMD, −0.29; (95% CI: −0.70 to 0.12); *p*-value = 0.7757] did not significantly decrease in the NAC-treated group compared to the placebo group (Fig. 2D and E, and Table 2). Three studies had data on inflammatory markers, including ferritin, and pooled analysis showed statistically insignificantly higher levels in the NAC-treated group than in the placebo group [SMD, 0.11; (95% CI: −0.27 to 0.48); *p-*value = 0.5798] with a high degree of heterogeneity (*I*^2^ = 73%) (Fig. 2C and Table 2).

**Table 2.** A table summarizing the number of NAC-treated studies on COVID-19 severity and mortality parameters, meta-analysis summary effect, 95% confidence interval, statistical significance, heterogeneity (%), and study bias.

COVID-19 severity indices	Number of studies	Summary effect	95% CI	*p*-value (Summary effect)	heterogeneity (*I*^2^, %)	*p*-value (Egger’s test)
Mortality	6	RR, 0.65	0.56 to 0.75	<0.0001	71.6	0.86
CRP	5	SMD, −0.32	−0.56 to −0.09	0.0070	87.6	0.28
D-dimer	3	SMD, −0.35	−0.59 to −0.10	0.0062	89.6	0.79
Ferritin	3	SMD, 0.11	−0.27 to 0.48	0.5798	72.7	0.31
Hospital stay	2	SMD, −0.06	−0.32 to 0.20	0.6394	62.1	----
ICU-stay	2	SMD, −0.04	−0.30 to 0.22	0.7757	59.3	-----
PaO_2_/FiO_2_	4	SMD, 0.76	0.48 to 1.03	<0.0001	90.9	0.86

### Therapeutic efficacy of NAC in reducing mortality of patients with COVID-19

**Figure 3.**
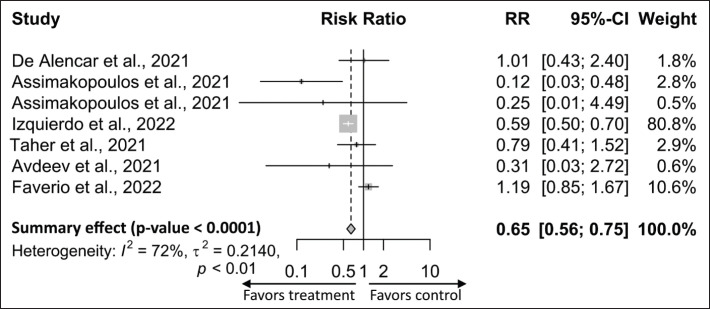
Forest plot for mortality from COVID-19 in the NAC treatment group versus the control group. Treatment, NAC; RR.

In this meta-analysis, mortality was the most meaningful outcome of NAC therapeutic efficacy in patients with COVID-19, and six studies had data on mortality (Tables 1 and 2). Pooled analysis indicated that mortality was significantly reduced in the NAC-treated group of patients with COVID-19 compared to the placebo group [RR, 0.65; 95% CI: 0.56 to 0.75; *p-*value 0.0001], though studies have a high degree of heterogeneity (*I*^2^ = 72%) (Fig. 3 and Table 2).

## Discussion

A meta-analysis demonstrated that the main risk factors for severe COVID-19 are increased age, male sex, diabetes, obesity, and hypertension [45]. Accumulating evidence suggests that these risk factors are associated with the severity and mortality of COVID-19 [46,47]. These risk factors also appear to contribute to increased oxidative stress, leading to increased COVID-19 severity and mortality [48–50]. The present study analyzed the therapeutic efficacy of NAC in reducing COVID-19 severity in terms of CRP, D-dimer, ferritin, PaO_2_/FiO_2_, duration of hospital and ICU stays, and mortality. The results suggested that NAC may reduce severity and mortality in patients with COVID-19; however, there was a high level of heterogeneity across studies (*I^2^* = 59%–90%) ( and ). In a recent meta-analysis by Paraskevas et al. [51], analyzing the effect of NAC in hospitalized COVID-19 patients, observational studies trended toward favorable outcomes in patients receiving NAC, but RCTs showed that pooled effects were close to the line of no effect. Our meta-analysis included eight studies, two RCTs, and six non-randomized trials with 20,503 participants, including 2,852 in the NAC-treated group and 17,651 in the placebo group analyzed together, which is another dimension of the study by Paraskevas et al. [51]. The pooled effect had a favorable outcome in reducing severity and mortality among patients receiving NAC. Another meta-analysis looking at the therapeutic efficacy of NAC in patients with ARDS caused by non-COVID-19 disease, similar to our results, reduced the duration of hospital and ICU stays; however, it did not significantly contribute to reduced mortality [52]. As part of their criteria and rationale, ROS scavenging by antioxidants has been used in clinical practice, and NAC is a widely used antioxidant. This may account for the difference in mortality between our study and Zhang et al. [52]. Furthermore, differences in causative agents, sample size, and underlying comorbidities may account for the differences between the present study and the study by Zhang et al. [52]. However, our study showed a reduced level of inflammatory markers called D-dimer and CRP and increased oxygenation (PaO_2_/FiO_2_ ratio), indicating that NAC may positively affect COVID-19 severity.

The limitations of the present study are the small number of studies included. Moreover, the studies have small numbers of events that may underpower the manuscript. This meta-analysis contained a mixture of observational, case-control, and RCT data, and there may be some concern about the risk of bias in the included studies. As the number of studies was less than 10, we could not perform funnel-plot analysis, and NOS and Egger’s tests were performed to determine study bias. Egger’s test *p*-value was greater than 0.05 for all considered indicators, indicating that studies were unbiased despite high levels of heterogeneity. The results of the present meta-analysis suggested that NAC may be a potent therapeutic for patients with COVID-19, especially in patients with underlying comorbidities. We also performed a comprehensive review using the relevant literature to validate the findings.

### Potential role of NAC in respiratory infections

An RCT published in 1997 looked at a total population of 262 people of both sexes in more than 20 different centers in Italy and treated them to either a placebo or a 600 mg NAC tablet twice daily for 6 months, and they looked at how many people came down with H1N1 flu [53]. There was no difference in that both groups had the same amount of flu. Still, when they looked at which one had symptoms of the flu, 79% of those in the placebo group came down with symptoms of the flu compared to only 25% of those with NAC treatment; the absolute risk reduction in those with NAC treatment was 0.5. Although this drug did not reduce influenza, it significantly decreased the incidence of clinical symptoms of the disease. They concluded that the treatment with NAC during the winter season significantly reduces influenza-like illness, particularly in elderly individuals with comorbidities. Another article published in 2010 showed that NAC inhibited viral replication and reduced inflammatory cytokines and suggested that antioxidants such as NAC represent a powerful additional treatment option that can be considered in the influenza pandemic [54]. A meta-analysis reported in 2017 looked at the efficacy of NAC treatment in ARDS and found a significant difference in the duration of ICU stay but no difference in mortality [52]. Study numbers were small, and additional studies are needed to provide sufficient evidence for the efficacy of NAC in ARDS, particularly mortality. A key finding was that no severe adverse reactions occurred in patients.

In a subsequent study, an RCT of NAC in community-acquired pneumonia (CAP) published in 2018 looked at the levels of oxidative markers and found that total antioxidant capacity (TAOC), malondialdehyde (MDA), SOD, and TNF α was similar between groups before treatment. Still, plasma levels of TNF-α and MDA decreased more in the NAC-treated group than in the non-NAC group with TAOC [55]. Again, they stated that no NAC-related adverse effects were found. Of course, this is for CAP, not COVID-19. It may be that oxidative stress plays a greater role in COVID-19 than in CAP. It might play a great role because this oxidative stress may lead to thrombosis in a hypercoagulable state due to the vWF, as we have previously described [9]. NAC with COVID-19 may open up another new front here in that NAC can affect not only the oxidative stress aspect of COVID-19 but also the hypercoagulable state set by the excess of the vWF.

### Potential role of NAC in COVID-19 infection

As mentioned above, since oxidative stress is a central mechanism of COVID-19 pathology, severity, and mortality, NAC was proposed to be administered to high-risk individuals with severe COVID-19 [56]. The first descriptive evidence of NAC efficacy in COVID-19 clinical cases was an observational study by Ibrahim et al. [32]. Intravenous administration of NAC showed clinical upliftment in patients over 40 with COVID-19 on mechanical ventilation, mainly through significant reductions in inflammatory markers, ferritin, and CRP [32]. Two grams of oral or IV GSH, a precursor to NAC therapy, has effectively reduced dyspnea in COVID-19 patients [57]. Later on, in a large cohort study of 19,208 hospitalized COVID-19 cases, 2,071 of whom were treated with 600 mg/kg oral NAC, mortality was significantly reduced [34].

Similarly, another two-center cohort study of 82 COVID-19 patients reported lower rates of respiratory failure, need for mechanical ventilation, and death when receiving 1,200 mg/day NAC [41]. In a case study, 24 COVID-19 patients were treated with IV NAC at a daily dose of 1,200 to 1,800 mg/kg, and 22 patients were in the control group [33]. NAC treatment significantly improved blood oxygenation (PaO_2_/FiO_2_ ratio) and decreased CRP and length of hospital stay. However, in an RC trial study in which 67 severe COVID-19 cases were given NAC 300 mg/kg for 20 h and 68 were given 5% dextrose, there was no difference in the duration of ICU and invasive mechanical ventilation stays or mortality between the two groups [35]. In a pilot study, 92 cases of ARDS associated with COVID-19 administered IV at a dose of 40 mg/kg found no difference in mortality between the group treated with NAC and the control [42]. Compared to the studies mentioned above, a high dose of NAC with a very short administration time was found to be highly positive. In an RCT that enrolled 46 patients with COVID-19-associated pneumonia, the NAC (1,200–1,500 mg IV)-treated group showed a significant increase in blood oxygen saturation and CRP values and a decrease in hospital stay [36].

Because of insufficient RCTs, several trials are ongoing to investigate the pharmacological efficacy of NAC in patients with COVID-19. For example, the NCT04792021 clinical trial evaluated the pharmacological efficacy of NAC (600 mg twice daily) on oxidative stress, TNF-α, and complications in patients with COVID-19 [58]. Another NCT 04455243 clinical trial, enrolling 1,180 participants, evaluated the efficacy of NAC (150 mg/kg every 12 h for 14 days, oral/IV ) in managing COVID-19 patients underlying comorbidities [59]. Recovery time is the primary outcome measure. The NCT04419025 clinical trial evaluated the efficacy of oral NAC in preventing severe COVID-19 pathogenesis. The objective of the trial has almost been completed, but no results have been posted yet [60].

NAC has been widely available, inexpensive, safe, and routinely used in clinical practice for many years. NAC administered orally or intravenously can suppress SARS-CoV-2 replication and improve outcomes when used immediately after the onset of signs and symptoms of COVID-19 [35,61]. Recommendations are that oral administration of NAC, as a prophylactic measure, can prevent a mild form of COVID-19 and that IV administration in the hospital can prevent severe morbidity, ICU admission, and mortality. Table 3 shows the recommended doses of NAC (oral and IV administration) for preventing and treating COVID-19 infection. Administration of NAC with another antiviral drug can dramatically reduce hospitalization, mechanical ventilation, and mortality [56]. Furthermore, it has been shown that copper exhibits strong antiviral effects if it is combined with NAC to reduce the viral load in the early stages of COVID-19 [62].

**Table 3.** Summary of recommended doses of NAC for prevention and treatment of COVID-19.

Clinical signs and symptom	Doses	Route of administration	References
Prevention			
Mild	600 mg twice daily	Oral	[56,63]
Treatment			
Mild	600 mg three time a day	Oral	[34]
Moderate	300 mg three time a day	Intravenous	[64]
Severe	300 mg 5–6 time a day	Intravenous	[36]

### Potential pharmacological mechanisms of NAC in COVID-19

Given that NAC exhibits direct and indirect antioxidant activities. It scavenges ROS by interacting with a free thiol as a direct antioxidant [65]. As a precursor of NAC, GSH participates in redox reactions that donate electrons during the detoxification of ROS as an indirect effect [66]. Decreased levels of GSH and thiols increase SARS-CoV-2 viral replication, and increasing viral load increases oxidative damage to vital organs such as the lungs and heart [67]. Moreover, the depletion of GSH triggers an apoptotic cascade in lymphocytes leading to lymphopenia, which is directly related to severe disease and high mortality, particularly in COVID-19 patients [68–70]. A recent study suggested that GSH deficiency is associated with increased levels of IL 1β, IL6, TNF-α, CRP, and D-dimer as a potential cause of increased susceptibility to COVID-19 infection, especially in elderly individuals with underlying comorbidities, such as diabetes, hypertension, and obesity [10].

Conversely, GSH treatment has been found to reduce viral infection and viral load, inhibit pro-inflammatory cytokine production (e.g., IL6, IL8, and TNFα), oxidative stress, and thrombosis, as well as potentially enhance immune function [10]. In addition, GSH supplementation has been found to modulate immune cells, enhancing both innate and adaptive immunity against SARS-CoV-2 infection [67], suggesting that GSH-enhancing therapy may be a cornerstone in reducing the severity and fatal outcome of COVID-19 [71]. Overall, the correlation between COVID-19/respiratory tract infection and GSH deficiency from a biochemical perspective is shown in Table 4.

NAC has been found to inhibit NF-κβ activation in respiratory syncytial and influenza viral infections [28], and NF-κβ activation is associated with increased expression of inflammatory cytokines such as IL-1β, IL-6, and IL-6. -8, IL-17, CRP, D-dimer, and TNFα in COVID-19 infection [72]. Furthermore, NAC has been found to restore the immune response, control inflammation, protect against thrombotic conditions, and exert an antiviral effect [27]. An experimental study showed that NAC protects against the detrimental effects of Ang II by inhibiting ACE2 [73].

**Table 4.** Biochemical associations between COVID-19 and GSH deficiency. Data compiled from [10,27,67,72,73].

Parameters	COVID-19/respiratory infection	GSH deficiency
Ang II	Increased	Increased
Ang 1,7	Decreased	Decreased
IL 1β, IL6	Increased	Increased
TNF-α	Increased	Increased
NF-κβ activation	Increased	Increased
CRP	Increased	Increased
D-dimer	Increased	Increased
Oxidative stress/ROS	Increased	Increased
Cytokine storm	Increased	Increased
Coagulability	Increased	Increased
Innate and adaptive immunity	Decreased	Decreased

**Figure 4.**
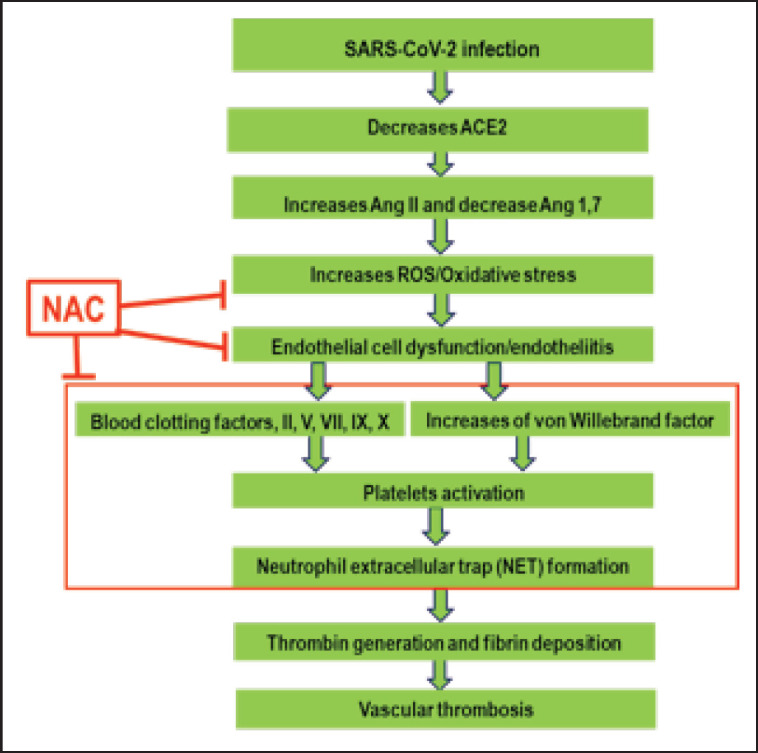
A schematic diagram summarizing the pathogenesis of SARS-CoV-2 infection and potential mechanisms of NAC in preventing COVID-19 infection.

In addition to antioxidant properties, NAC inhibits blood coagulation and exerts a thrombolytic effect by reducing the level of disulfide bonds (-S-S-) to sulfhydryl (-SH) groups in vWF polymers, resulting in vWF fragmentation and subsequent platelet dissociation [74,75]. Consistent with these, NAC has inhibited ROS and coagulation factor production in laboratory animals [52,55,57,74]. We summarize the pathogenesis of COVID-19 and the potential mechanisms of NAC that may be beneficial in inhibiting COVID-19 infection (Fig. 4).

The SARS-CoV-2 virus binds to the ACE2 receptor on the cell membrane, which reduces the level of ACE2. The suppression of ACE2 decreases Ang 1−7 synthesis and increases Ang II levels, which favors oxidative stress. Oxidative stress induces endotheliitis and endothelial cell dysfunction, leading to the activation of blood coagulation factors such as factors II, V, VII, IX, and X, as well as the vWF released from the sub-endothelial space. The blood coagulation cascade and vWF activate platelets, interacting with neutrophils and stimulating NET formation.

NETs stimulate thrombin generation and fibrin deposition, leading to vascular thrombosis and contributing to disease severity and mortality.

NAC can act on ROS to reduce oxidative stress and restore the endothelium from endotheliitis, thereby reducing the amount of vWF released from the sub-endothelium that inhibits platelet activation and NET formation and ultimately prevents vascular thrombosis and COVID-19 infection and severity.

## Conclusion

Continuous mutation of the SARS-CoV-2 virus threatens the efficacy of current COVID-19 vaccines and therapeutics such as monoclonal antibodies and antiviral drugs, thus warranting other treatment options targeting oxidative stress and cytokine storm. Recent clinical studies have suggested that, besides vaccines, NAC may be a potential therapy for patients with COVID-19, particularly for patients with comorbidities. This meta-analysis indicates that NAC may reduce COVID-19-associated complications and mortality. As the number of studies with certainty was very low, more research is needed to confirm that NAC is effective and safe in patients with COVID-19. However, this comprehensive review and meta-analysis points to potential research gaps and a better understanding of the efficacy of NAC that may pave the way for mitigating COVID-19-related complications and reducing mortality, and these data will be valuable resources for healthcare professionals, policymakers, and researchers.

## List of Abbreviations

ACE2, angiotensin-converting enzyme 2; ARDS, acute respiratory distress syndrome; CAP, community-acquired pneumonia; COVID-19, coronavirus disease 2019; CRP, C-reactive protein; FDA, Food and drug administration; ICU, intensive care unit; MDA, malondialdehyde; NAC, N-acetylcysteine; NADPH, nicotinamide dinucleotide phosphate; PaO_2_/FiO_2_ ratio, oxygenation; PRISMA, preferred reporting items for systematic reviews and meta-analysis; RCT, Randomized control trial; ROS, reactive oxygen species; RR, risk ratio; SARS-CoV-2, Severe acute respiratory syndrome coronavirus-2; SMD, standardized mean difference; SOD, superoxide dismutase; TAOC, total antioxidant capacity; TNF-α, tumor necrosis factor- α.
